# A novel form of docetaxel polymeric micelles demonstrates anti-tumor and ascites-inhibitory activities in animal models as monotherapy or in combination with anti-angiogenic agents

**DOI:** 10.3389/fphar.2022.964076

**Published:** 2022-08-24

**Authors:** Leilei Guo, Xiaokang Qin, Liting Xue, Janine Y. Yang, Yumei Zhang, Shunwei Zhu, Gang Ye, Renhong Tang, WenQing Yang

**Affiliations:** ^1^ State Key Laboratory of Translational Medicine and Innovative Drug Development, Jiangsu Simcere Pharmaceutical Co. Ltd, Nanjing, China; ^2^ Massachusetts Eye and Ear, Harvard Medical School, Boston, MA, United States; ^3^ Department of Oncology, Shanghai East Hospital, School of Medicine, Tongji University, Shanghai, China

**Keywords:** docetaxel polymeric micelles, anti-tumor effects, ascites-inhibitory activities, anti-angiogenic agents, combination therapy

## Abstract

Malignant ascites (MA) is caused by intraperitoneal spread of solid tumor cells and results in a poor quality of life. Chemotherapy is a common first-line treatment for patients with MA. Taxotere ^®^ (DTX) is widely used in solid tumor therapies. However, the low water solubility and side effects caused by additives in the formulation restrict the clinical application of docetaxel. HT001 is a clinical stage docetaxel micelle developed to overcome the solubility issue with improved safety profiles. To support clinical development and expand clinical application of HT001, this study used *in vitro* and *in vivo* approaches to investigate the anti-tumor effects of HT001 when applied as monotherapy or in combination with anti-angiogenic agents. HT001 demonstrated comparable anti-proliferative activities as docetaxel in a broad range of cancer cell lines *in vitro*. Furthermore, HT001 suppressed tumor growth in a dose-dependent manner in A549, MCF-7, and SKOV-3 xenograft tumor mouse models *in vivo*. In a hepatocellular carcinoma H22 malignant ascites-bearing mouse model, HT001 presented a dose-dependent inhibition of ascites production, prolonged animal survival, and reduced VEGF levels. When dosed at 20 mg/kg, the HT001-treated group exhibited curative results, with no ascites formation in 80% of mice at the end of the study while all the mice in the vehicle control group succumbed. Similar results were obtained in HT001 treatment of mice bearing malignant ascites produced by human ovarian cancer ES-2 cells. Notably, the combination of HT001 with Endostar not only significantly reduced ascites production but also prolonged survival of H22 ascites-bearing mice. HT001 showed similar PK and tissue distribution profiles as DTX in non-rodent hosts. Collectively, these results demonstrate potent anti-tumor activity of HT001 in multiple solid tumor models or malignant ascites models, and reveal synergistic effects with anti-angiogenic agents, supporting the clinical development and clinical expansion plans for HT001.

## Introduction

Malignant ascites (MA) is a pathological condition arising from a variety of abdominal neoplasms and significantly affects patient’s quality of life (Cavazzoni et al., 2013). Ovarian, endometrial, colorectal, gastric, pancreatic, and peritoneal malignancies are often associated with MA, and up to 15% of all patients with gastrointestinal cancers develop ascites at some stage of their disease (Smith and Jayson, 2003; Keen et al., 2010; Monavarian et al., 2022; Tokalioglu et al., 2022). Systemic therapy against the underlying malignancy is the mainstay of treatment, while additional measures are usually needed to help ascites control. MA represents an unmet medical need in the clinic.

Docetaxel (DTX) exhibits a broad spectrum of antitumor activities against various malignancies including non-small-cell lung cancer, ovarian cancer, and liver cancer (Hirte et al., 2021; Tian et al., 2021; Yamashita et al., 2021). DTX exerts its cytotoxic activity by stabilizing microtubule assembly, preventing microtubule depolymerization, and inhibiting metaphase-to-anaphase transition (Fujii, 2008; Mosca et al., 2021). Microtubules do not disassemble in the presence of DTX but rather accumulate in the cell and initiate apoptosis (Mosca et al., 2021). Despite its favorable antitumor effects, DTX has several limitations that have restricted its clinical use, including poor aqueous solubility and systemic toxicity due to nonspecific distribution (Zhang et al., 2016; Grudén et al., 2017; Tang et al., 2017; Lu et al., 2020). Meanwhile, the therapeutic efficacy of DTX is weakened by high hydrophobicity, short *in vivo* half-life, and dose-limiting severe side effects (Hennenfent and Govindan, 2006). DTX is currently formulated as the marketed product Taxotere® reconstituted in Tween 80 and anhydrous ethanol, which has been associated with severe adverse effects, including acute hypersensitivity reactions, cumulative fluid retention, neurotoxicity, and musculoskeletal toxicity (Mi et al., 2011). Conjugation of drugs to polymers has been proven to be a successful method to improve drug solubility and therapeutic property (Xu et al., 2009; Jinturkar et al., 2012; Siegel, 2014; Song et al., 2014; Crucho, 2015). Studies have been carried out to improve the solubility, distribution and potential therapeutic effects of DTX, including physical entrapment of DTX into nano-vectors such as micelles (Tan et al., 2017; Zhang et al., 2022). Various forms of nano-formulated DTXs have been developed and characterized, including DTX-mPEG-PDLLA from laboratory of our collaborator (Zhang et al., 2022) and DTX-MPEG-PDLLA-PLL from others (Tan et al., 2017).

The detailed MOA and disease biology underling MA are not completely understood. Systemic symptoms-reducing modalities and chemotherapies are commonly used as first-line treatments for MA patients. However, limited efficacy and a lack of long-lasting benefit represent biggest challenges in the clinic, especially for patients with recurrent ascites. Linkages between enhanced angiogenesis and malignant ascites secretion have been made or suggested. Vascular endothelial growth factor (VEGF) is overexpressed in a variety of tumors causing malignant ascites, and markedly elevated concentrations of VEGF have been found in malignant pleural effusions and ascites derived from solid tumor patients (Filali et al., 2012; Yu et al., 2013; Hui and Meng, 2015; Monk et al., 2016; Angelucci et al., 2018; Paolino et al., 2019; Patel and Cecchini, 2020; Zhao et al., 2021; Chilimoniuk et al., 2022). In addition, serum concentrations of soluble VEGF are elevated in patients with various solid tumors, positively correlating with disease stage (Luo et al., 1998; Yoneda et al., 1998; Das, 2006; Gaya and Tse, 2012; Smolle et al., 2014; Monk et al., 2016; Ford et al., 2020; Zhao et al., 2021). A recent clinical research showed that intraperitoneal administration of recombinant human endostatin improved efficacy and survival for gastric cancer with ascites (Zhan et al., 2022), implying anti-angiogenic may be a new effective therapy for the treatment of MA. Endostar is a recombinant human endostatin exerting anti-angiogenic effects via VEGF-related signaling pathways, which has been shown to suppress the growth of various human cancers by inhibiting neovascularization *in vivo* (Liang et al., 2016). Endostar was approved by the National Medical Products Administration (NMPA) in 2005 for the treatment of NSCLS (Fu et al., 2011). In recent years, combination therapy has been proven to achieve better therapeutic efficacy in various type of cancer patients. The exploration of combining anti-angiogenic agents with chemotherapeutic drugs formulated in nano-particles may represent a great value in perusing improved therapeutic activities for MA patients.

To attempt to address concerns and questions raised above, this paper was aimed to 1) develop and characterize a novel form of DTX polymeric micelle HT001; 2) determine HT001’s *in vivo* anti-tumor activities in preclinical models; and most importantly 3) test feasibility of treating MA using a combinatorial approach engaging both HT001 and the anti-angiogenic agent Endostar. The results of this study may provide new therapeutic directions or options for MA patients and support the clinical development plans for HT001 and Endostar.

## Materials and methods

### Chemicals and reagents

Endostar was obtained from Jiangsu Simcere Pharmaceutical Co., Ltd (China). Docetaxel injection (Taxotere®) was purchased from Sanofi-Aventis Deutschland GmbH (Germany) and Cisplatin (CDDP) was purchased from Jiangsu Hengrui Pharmaceuticals Co., Ltd (China). Docetaxel injection was configured as a mother liquor according to the instructions, and then diluted with normal saline to the corresponding working solution concentration. Others were all diluted in normal saline.

### Preparation and characterization of docetaxel polymeric micelles HT001

Docetaxel polymeric micelles HT001 was synthesized using protocols described by Zhang et al. (2022). Briefly, mPEG-PDLLA (480 mg) and DTX (20 mg) were co-dissolved in methanol, mixed, and heated over 47°C rotary steam for at least 1 h to remove the solvent. Normal saline was added and hydrated at 60°C to prepare HT001. The HT001 solution was obtained, filtered, sterilized, and lyophilized to obtain a lyophilized preparation of HT001.

The morphology of HT001 was observed by transmission electron microscopy (TEM, HT7700 Exalens, Hitachi, Japan). One drop of micelles was dispensed on the 300-mesh copper grid, and the excessive liquid was removed using filter paper. The DTX PMs were negatively stained with 3% (*w/v*) phosphotungstic acid and dried at room temperature before observation.

### Cell culture

The human breast cancer cell line MCF-7 was purchased from the American Type Culture Collection (ATCC) and maintained in Minimum Essential Medium Non-Essential Amino Acids (MEM-NEAA) medium (HyClone, United States) containing 10% fetal bovine serum (FBS) (Gibco, United States) and 1% penicillin-streptomycin (P/S) (Gibco, United States). The human breast cancer cell line MDA-MB-231 was purchased from Shanghai Institute of Biological Sciences, Chinese Academy of Sciences, and maintained in Roswell Park Memorial Institute (RPMI) 1640 medium (Gibco, United States) containing 10% FBS and 1% P/S. The human breast cancer cell line MDA-MB-435, human non-small cell lung cancer cell lines A549 and H460, and human ovarian cancer cell lines SKOV-3 and ES-2 were purchased from Institute of Basic Medical Sciences, Chinese Academy of Medical Sciences, and maintained in RPMI 1640 medium containing 10% FBS and 1% P/S. The human embryonic lung fibroblast cell line MRC-5 was purchased from Institute of Basic Medical Sciences, Chinese Academy of Medical Sciences, and maintained in MEM-NEAA (Gibco, United States) containing 10% FBS and 1% P/S. Mouse hepatoma cell line H22 was purchased from Nanjing Cobioer Biosciences CO., LTD (Nanjing, China) and maintained in RPMI 1640 medium containing 10% FBS and 1% P/S. The cell lines mentioned above were in their passages 10–15 during the experiments.

### Cell viability assay

A549, H460, MCF-7, MDA-MB-231, MDA-MB-435, SKOV-3, or MRC-5 cell suspension was seeded in a 96-well flat-bottom tissue culture plate with 5000 cells per well. After 72-h incubation with HT001 or DTX, cell viability was assessed by Cell Counting Kit (CCK)-8 assay. Specifically, the culture medium was replaced with 100 μL fresh medium containing 10 μL CCK-8 solution (Dojindo Laboratories, United States), and cells were incubated at 5% CO_2_ in a humidified incubator at 37°C for 0.5–1.5 h. The absorbance at a wavelength of 450 nm was detected by a SpectraMax M5 microplate reader (Molecular Devices, United States). The IC_50_ value was calculated based on the luminescence at different concentrations by GraphPad Prism 8.0 (GraphPad Software Inc., San Diego, CA, United States).

### Laboratory animals

Female Balb/c mice, Balb/c nude mice, and CB-17 SCID mice at 6–8 weeks were all purchased from Shanghai Lingchang Biotechnology Co., Ltd (Shanghai, China). Eight-week-old Sprague-Dawley (SD) rats were purchased from Beijing Vital River Laboratory Animal Technology Co., Ltd. (Beijing, China). Beagle dogs (male/female) weighing 7.73–8.37 kg at about 6–8 months old were purchased from Mars Biological Technology Co., Ltd (Beijing, China). All experimental procedures involving animals and their care were conducted in accordance with the State Council Regulations for Laboratory Animal Management (Enacted in 1988) and were approved by the Institutional Animal Care and Use Committee of the Jiangsu Simcere Pharmaceutical Co. Ltd.

### Efficacy studies in A549, MCF-7, and SKOV-3 xenograft mouse models

To establish A549, MCF-7, and SKOV-3 xenograft mouse models, 5^∗^10^6^ A549, MCF-7, and SKOV-3 cells per mouse were injected subcutaneously into the right flank of female CB-17 SCID mice, Balb/c nude mice, and Balb/c nude mice respectively. Mice were sacrificed when tumor volume reached 300 mm^3^. Tumors were collected and divided into small pieces (2^∗^2^∗^2 mm). The resulting small tumor pieces were implanted subcutaneously into the right flank of mice. As average tumor volume reached 100–300 mm^3^, the mice were randomly assigned to experimental groups according to the tumor volume and treated with normal saline, HT001 (5, 10, 20, or 40 mg/kg), or DTX (5, 10, 20, or 40 mg/kg) intravenously. The tumor volume and the bodyweight of the animals were measured twice a week. The long diameter a) and the short diameter b) of the tumor were measured using a caliper, and the tumor volume was calculated using the following formula: V = 0.5^∗^a^∗^b^2^.

### Efficacy studies in the H22 and ES-2 ascites mouse models

To establish the H22 ascites mouse model, Balb/c mice were intraperitoneally injected with 1^∗^10^7^ cells H22 cells. After 7 days, the ascites cells were harvested from mice and re-suspended in saline and each naïve Balb/c mouse was injected intraperitoneally with 5^∗^10^5^ ascites cell suspension. Three days after H22 tumor cells inoculation, the mice were randomly assigned to experimental groups (*n* = 16) according to the bodyweight, and treated with normal saline, CDDP (5 mg/kg), HT001 (1.5, 5, or 20 mg/kg), Endostar (8 mg/kg), or combination intraperitoneally. The bodyweight of the animals was measured three times a week and the survival of mice was monitored daily. After 8 days of treatment, 6 mice from each group were sacrificed to collect plasma and ascites for analysis. The weights of ascites were recorded, and cells with ascites were counted. In addition, VEGF levels in plasma and ascites were measured by Quantikine™ mouse VEGF immunoassay kit (R&D Systems, United States). The remaining mice were used for monitoring survival.

To establish the ES-2 ascites mouse model, Balb/c nude mice were intraperitoneally injected with 1^∗^10^7^ cells ES-2 cells. Seven days after ES-2 cells inoculation, the mice were randomly assigned to experimental groups (*n* = 16) according to the bodyweight and treated with normal saline, CDDP (5 mg/kg), or HT001 (5 or 20 mg/kg) intraperitoneally. The bodyweight of the animals was measured three times a week and the survival of mice was monitored daily. After 7 days of administration, 6 mice from each group were sacrificed to collect plasma and ascites for analysis. The weights of ascites were recorded, and cells with ascites were counted. In addition, VEGF levels in plasma and ascites were measured by Quantikine™ mouse VEGF immunoassay kit (R&D Systems, United States). The remaining mice were used for monitoring survival.

### *In vivo* pharmacokinetic and tissue distribution studies

Eight-week-old Sprague-Dawley (SD) rats were intravenously administered with a single dose of 2.5, 5, 10 mg/kg HT001 or 5 mg/kg DTX (six rats per group, male: female = 1:1). Blood was collected from the jugular vein in heparinized tubes at 0.083, 0.25, 0.5, 1, 2, 3, 4, 6, 8, and 24 h after administration. Blood samples were centrifuged at 3,000 rotations per minute (rpm) for 10 min to obtain plasma and analyzed by LC-MS/MS (Waters Corp., Manchester, United Kingdom). Beagle dogs were intravenously administrated with a single dose of 1 mg/kg of DTX or HT001 (6 dogs per group, male: female = 1:1). Blood was collected from the forelimb vein in heparinized tubes at 0.25, 0.5, 0.55, 1, 2, 4, 6, 8, and 24 h after administration. Blood samples were centrifuged at 3000 rpm for 10 min to obtain plasma and analyzed by LC-MS/MS.

Biodistribution studies were performed in MCF-7 tumor-bearing Balb/c nude mice. After the tumor volume reached 100–300 mm^3^, mice were randomly assigned to two groups (24 mice per group, male to female ratio 1:1). Mice were intravenously administered with 10 mg/kg HT001 or DTX. At the various time points (0.5, 2, 6, and 24 h, *n* = 6, male: female = 1:1), mice were anesthetized and sacrificed. The plasma and major organs, including brain, heart, lung, liver, kidney, spleen, stomach, small intestine, fat, muscle, gonads, tumor, and marrow were harvested. Organs were weighed and homogenized with 400 μL of PBS/10 international units (IU) of heparin solution and extracted. The concentration of compounds was analyzed by LC-MS/MS.

### Tolerability and toxicology studies

Eight-week-old SD rats were intraperitoneally administered with normal saline, 5, 10, or 16 mg/kg HT001 once a week (ten rats per group, male: female = 1:1). The bodyweight and survival of rats were monitored during the experiments. On the day after the fifth dose, rats were sacrificed. The liver, kidney, and spleen samples from normal saline and 16 mg/kg HT001 groups were harvested and fixed in Zn fixing buffer for about 36 h at room temperature, changed to ddH_2_O for 5 min, dehydrated, and embedded in paraffin using the Leica ASP300S system and EG 1150H+C system (Leica, Wetzlar, Germany). The liver, kidney, and spleen sections were stained using Hematoxylin and Eosin method. Each stained slide was observed via a light microscope (Nikon Labophot, Japan) in a blind manner to evaluate histopathological changes in liver, kidney, and spleen.

### Statistical and data analysis

All results are expressed as the mean ± standard error of mean (SEM). The statistical analysis of data was performed using two-way ANOVA followed by Tukey’s multiple comparisons test in efficacy studies using GraphPad Prism 8 (GraphPad Software Inc., San Diego, CA, United States). Original spectrum concentration and accuracy were outputted using UNIFI (Waters, United States). The pharmacokinetic parameters were calculated using non-chamber model (NCA) method in WinNolin (V6.2) (Certara, Inc., United States). The student’s-t test was conducted to evaluate the statistical significance in pharmacokinetic studies and biodistribution assessment studies using Microsoft Office EXCEL (2007) (Microsoft, United States). *p*-values < 0.05 were considered statistically significant.

## Results

### Synthesis and characterization of HT001

**FIGURE 1:**
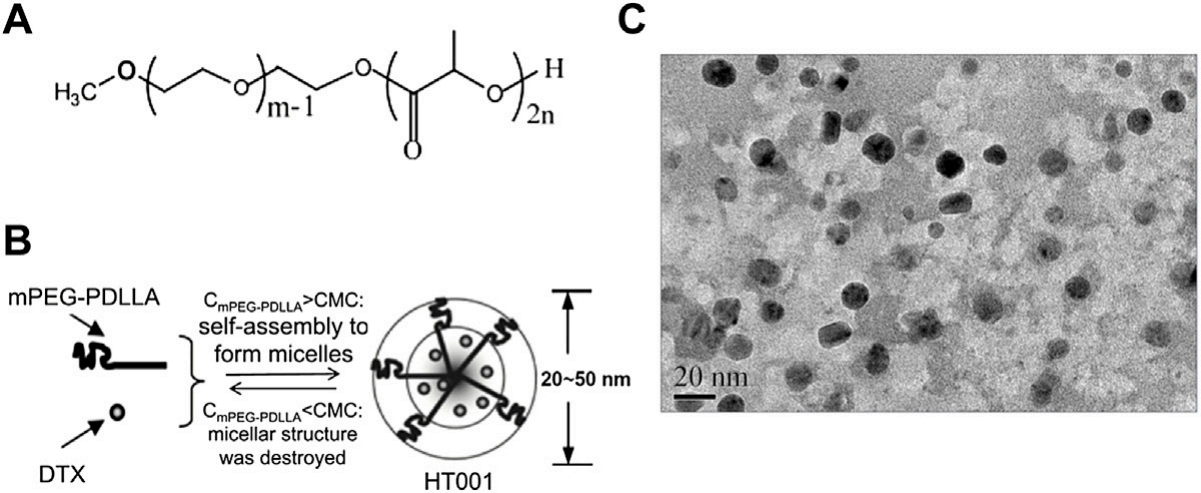
Synthesis and characteristics of mPEG-PDLLA and HT001. **(A)** The structure of polyethylene glycol monomethyl ether polylactic acid block polymer (mPEG-PDLLA). **(B)** The schematic process of HT001 synthesis. **(C)** Transmission electron microscopy image of HT001. The morphology of micelles was examined by transmission electron microscope.

The structure of polyethylene glycol monomethyl ether polylactic acid block polymer (mPEG-PDLLA) was shown in Figure 1A. The obtained copolymers were characterized by nuclear magnetic resonance, and the results reveal that the molecular weight is 3647 g/mol and the polydispersity coefficient value was 1.2 (data not shown). The synthesis schematic of HT001 was shown in Figure 1B. Lyophilized HT001 preparation was analyzed by transmission electron microscope. As shown in Figure 1C, polymer structures were approximately spherical and the micelles were formed. The solubility of HT001 in normal saline was 2.0 mg/ml. The basic chemical and structural features of HT001 (also known as DTX-mPEG-PDLLA) were characterized and the results published recently (Zhang et al., 2022). The drug loading efficiency, Zeta potential and DLS of HT001 were 4.27 ± 0.36%, −3.13 mV and 25 nm, respectively (Zhang et al., 2022). The result of drug release assay showed that less than 30% and 58% of HT001 was released in 6 and 72 h, respectively (Zhang et al., 2022). In comparison, DTX had a much faster release kinetics, e.g., ∼45% of DTX was released in the 1 h, and >90% of DTX was released in 6 h (Zhang et al., 2022).

### HT001 exhibits dose-dependent cytotoxicity in various cancer cells

**FIGURE 2:**
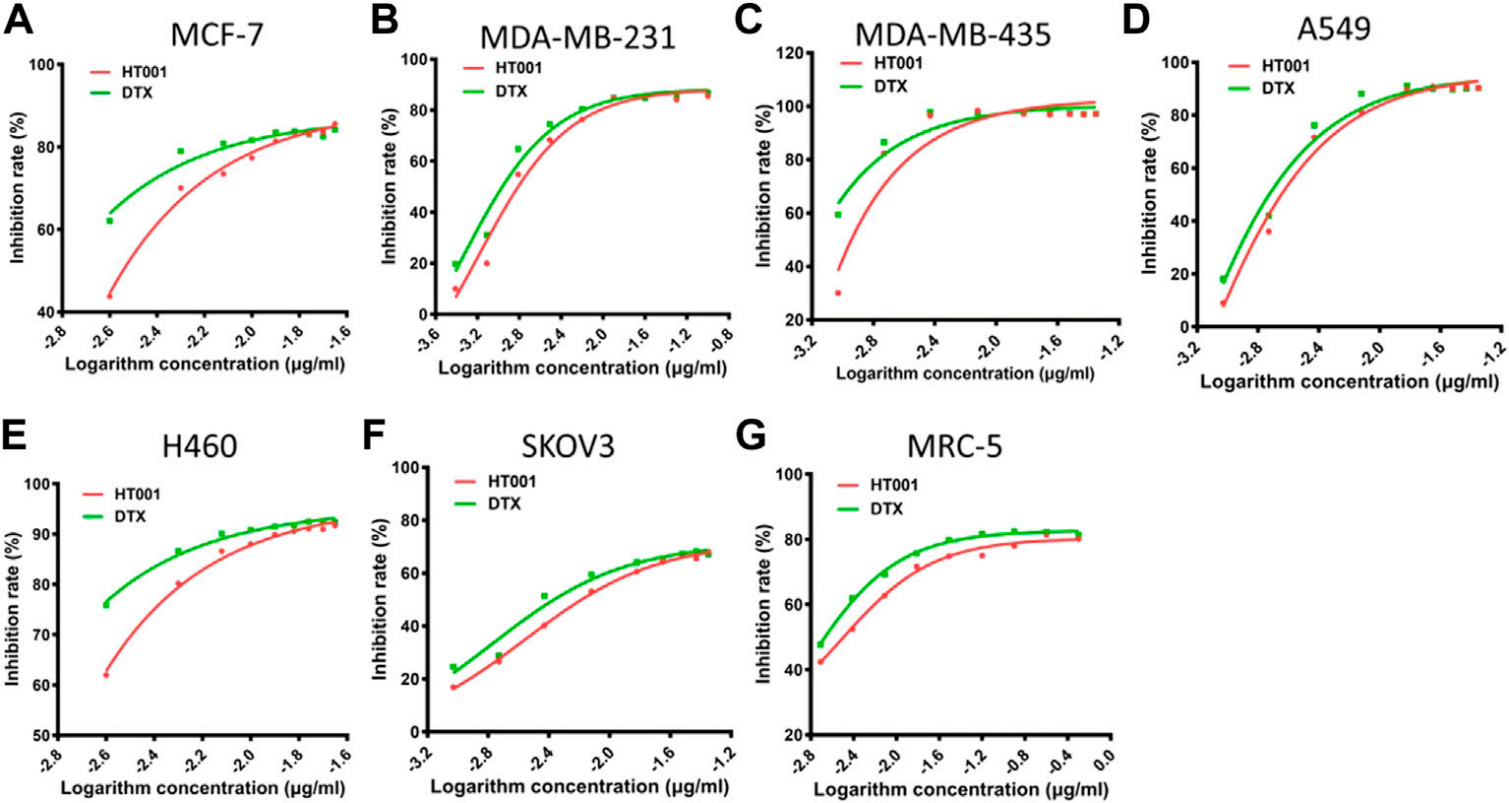
Cytotoxicity of HT001 and DTX *in vitro*. A cell suspension of 5000 cells/well was seeded in 96-well flat-bottom tissue culture plates. Cell viability was evaluated by CCK-8 assay after 72 h treatment with HT001 or DTX. **(A–G)** Cell viability inhibition in different cell lines.

Since DTX is effective in treating human non-small cell lung cancer, breast cancer, and ovarian cancer, human non-small cell lung cancer cells A549 and H460, human breast cancer cells MCF-7, MDA-MB-231, and MDA-MB-435, and human ovarian cancer cells SKOV-3 were chosen to study the cytotoxicity of HT001 using CCK-8 assay. The cytotoxicity of DTX and HT001 on human embryonic lung fibroblast cell MRC-5 was also studied. Upon treatment of DTX or HT001, cell viability decreased dose-dependently (Figures 2A–G). The IC_50_ values of HT001 and DTX in MCF-7, MDA-MB-231, MDA-MB-435, A549, H460, SKOV-3, and MRC-5 cells are listed in Table 1. These results reveal that comparable cytotoxic activities between HT001 and DTX are observed in majority of cell lines tested *in vitro* while in some cell lines (MCF-7, H460, and MDA-MB-435), HT001 exhibits weaker cytotoxicity than DTX.

**TABLE 1:** The IC_50_ values of HT001 and DTX in various cell lines.

Cell lines	MCF-7	MDA-MB-231	MDA-MB-435	A549	H460	SKOV-3	MRC-5
HT001(ng/mL)	2.6	2.2	1.1	3.0	1.3	10.2	2.2
DTX (ng/ml)	0.6	1.4	0.5	2.3	0.4	6.9	0.9

### HT001 suppresses tumor growth in A549, MCF-7 and SKOV-3 xenograft mouse models in a dose-dependent manner

**FIGURE 3:**
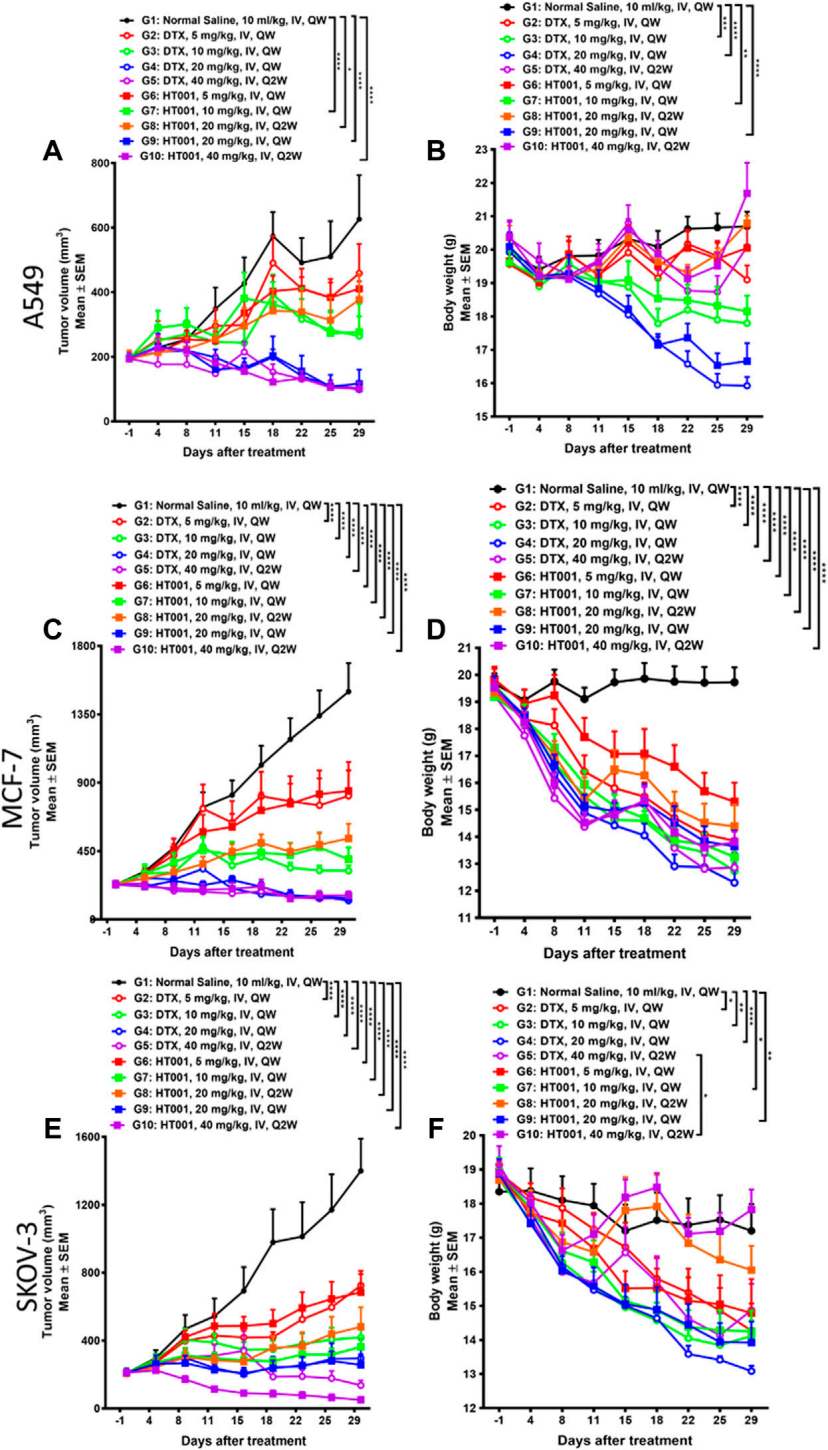
Anti-tumor effects of HT001 and DTX in A549, MCF-7, and SKOV-3 xenograft mouse models. A549, MCF-7, and SKOV-3 tumor-bearing mice were intravenously administrated with normal saline, DTX, or HT001 once a week or once every 2 weeks (*n* = 8/group). **(A,C,E)** Tumor growth curves of A549, MCF-7, or SKOV-3 tumor bearing mice after treatment (*n* = 8/group). **(B,D,F)** Bodyweight graphs of A549, MCF-7, or SKOV-3 tumor bearing mice after treatment. The error bars represent SEM. IV, intravenous; QW, once weekly; Q2W, once every 2 weeks; *, *p* < 0.05; **, *p* < 0.01; ***, *p* < 0.001; ****, *p* < 0.0001.

The anti-tumor effects of HT001 and DTX were also compared in the A549, MCF-7, and SKOV-3 xenograft mouse models. Compared with normal saline group, tumor growth was inhibited in a dose-dependent manner when treated with HT001 or DTX in three tumor mouse models, and generally the anti-tumor effect of HT001 was comparable or superior to DTX at the same dose and frequency (Figures 3A,C,E). Tumor photographs of all animals at end of studies are shown in Supplementary Figures S1A–C. Generally, tumor bearing mice presented a decrease in bodyweight at higher doses of HT001 or DTX, and Q2W dosing frequency resulted in a lower bodyweight loss than QW (Figures 3B,D,F). HT001 presented a lower animal bodyweight loss compared with DTX at same dose and dosing frequency. Notably, SKOV-3 tumor bearing mice treated with 40 mg/kg HT001 biweekly presented better anti-tumor efficacy and lower bodyweight loss than mice treated with 40 mg/kg DTX biweekly. This result indicates that the efficacy of HT001 is comparable to DTX while the safety of HT001 is superior than DTX, at least in some tumor mouse models that have been evaluated.

### HT001 prolongs the survival of ascites-bearing mice

**FIGURE 4:**
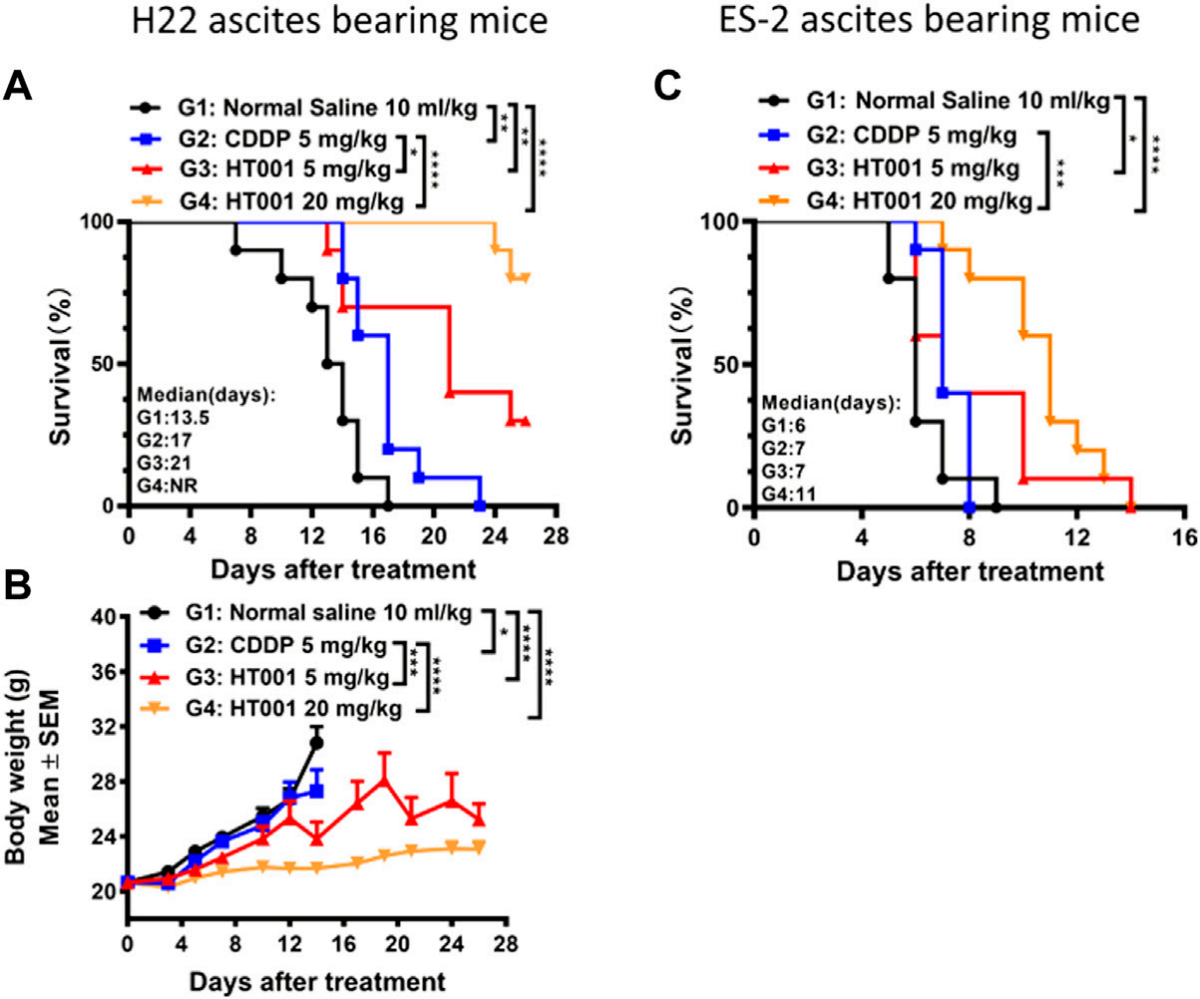
Effects of HT001 and CDDP on H22 and ES-2 ascites bearing mice. H22 or ES-2 ascites-bearing mice were intraperitoneally administrated with normal saline, 5 mg/kg CDDP, 5 mg/kg HT001, or 20 mg/kg HT001 (*n* = 16/group) once a week. After 7 or 8 days of administration, 6 mice from each group were sacrificed for analysis and the remaining mice were used for monitoring survival. **(A,C)** Kaplan-Meier survival analysis of H22 or ES-2 ascites-bearing mice. NR: not reached, more than 50% of mice survived at the end of the study and the medium survival time cannot be reached. **(B)** Bodyweight of H22 ascites -bearing mice. The bodyweight data points are not shown when there are less than 3 animals in a group. The error bars represent SEM. *, *p* < 0.05; **, *p* < 0.01; ***, *p* < 0.001; ****, *p* < 0.0001.

Malignant ascites is caused by intraperitoneal spread of solid tumor cells and results in a poor quality of life. Here, we further investigated the anti-tumor effects of HT001 in mouse models of malignant ascites to provide evidence for clinical development of HT001 to treat cancer patients with malignant ascites. Cisplatin (CDDP) is widely used in malignant ascites therapies. Thus, *in vivo* efficacies upon HT001 or CDDP treatment were compared in mouse hepatocyte cancer H22 and human ovarian cancer ES-2 ascites-bearing mice, respectively. Both CDDP and HT001 could prolong the survival of H22 ascites-bearing mice but HT001 showed a significantly better efficacy than CDDP at 5 mg/kg in both lifetime extension and bodyweight (reflects ascites weight) reduction (Figures 4A,B). In addition, a dose-dependent efficacy of HT001 was been observed. In a more aggressive ES-2 ascites mouse model, 5 mg/kg HT001 and 5 mg/kg CDDP showed comparable slight prolongation of mice survival while 20 mg/kg HT001 significantly prolonged the survival of ascites-bearing mice, compared with both normal saline group and 5 mg/kg CDDP group (Figure 4C). In summary, HT001 demonstrated dose-dependent activities in survival extension of ascites-bearing mice and HT001 was apparently more efficacious than CDDP.

### HT001 suppresses ascites formation in H22 and ES-2 ascites-bearing mice

**FIGURE 5:**
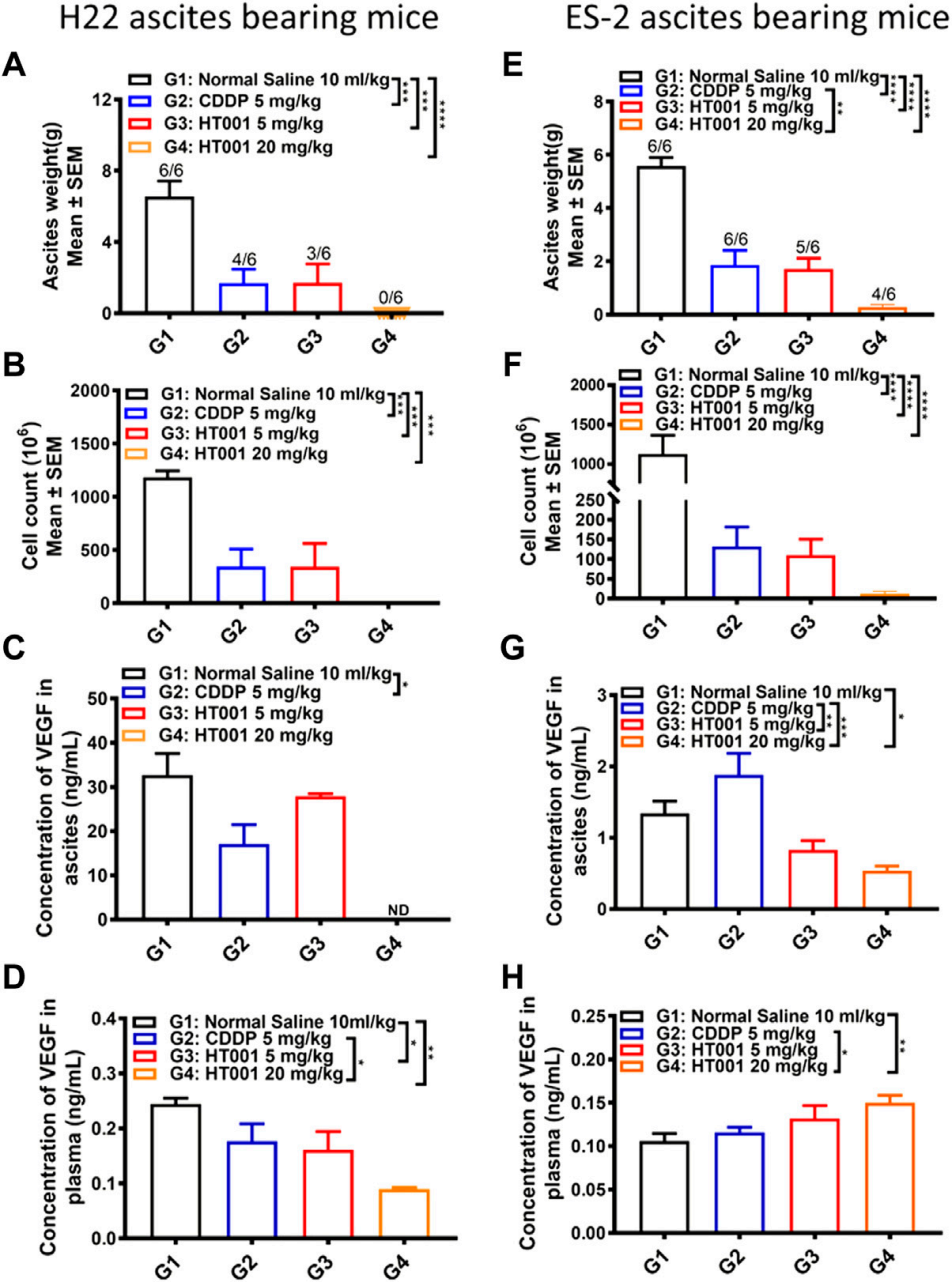
*Ex vivo* analysis of plasma and ascites samples from the H22 and ES-2 ascites-bearing mice. The ascites and plasma from H22 or ES-2 ascites bearing mice sacrificed 7 or 8 days after treatment as in Figure 4 were analyzed. **(A,E)** Ascites weight of H22 and ES-2 ascites-bearing mice. The numbers on the column represents number of mice with ascites formed/all analyzed mice. **(B,F)** Cell counts in ascites from H22 and ES-2 ascites bearing mice. **(C,G)** VEGF levels in ascites from H22 and ES-2 ascites-bearing mice. **(D,H)** VEGF levels in plasma from H22 and ES-2 ascites-bearing mice. Error bars represent SEM. *, *p* < 0.05; **, *p* < 0.01; ***, *p* < 0.001; ****, *p* < 0.0001.

To analyze the effects of HT001 on ascites formation, 6 ascites-bearing mice from each group were sacrificed 8 or 7 days after treatment in H22 or ES-2 ascites mouse model shown in Figure 4. As shown in Figures 5A,B, treatment of either CDDP or HT001 significantly reduced the ascites weight and cell counts compared with normal saline group in the H22 ascites mouse model. Notably, there was no ascites observed in all the mice analyzed after 8 days treatment of 20 mg/kg HT001. Similar results were observed in ES-2 ascites mouse model (Figures 5E,F).

The VEGF levels in plasma and ascites from H22 or ES-2 ascites-bearing mice after CDDP or HT001 treatment were measured by ELISA. Both CDDP and HT001 reduced the VEGF levels in plasma and ascites from H22 ascites-bearing mice (Figures 5C,D). Only HT001 but not CDDP reduced the VEGF levels in ascites from the more aggressive ES-2 ascites-bearing mice (Figure 5G). No reduction of VEGF levels was observed in plasma from ES-2 ascites-bearing mice upon CDDP or HT001 treatment (Figure 5H).

Together, the results demonstrate that HT001 dose-dependently inhibited ascites formation and reduced VEGF levels in H22 and ES-2 ascites-bearing mice, correlating with prolonging survival in those two ascites mouse models.

### HT001 synergizes with anti-angiogenic agent endostar in the H22 ascites mouse model

**FIGURE 6:**
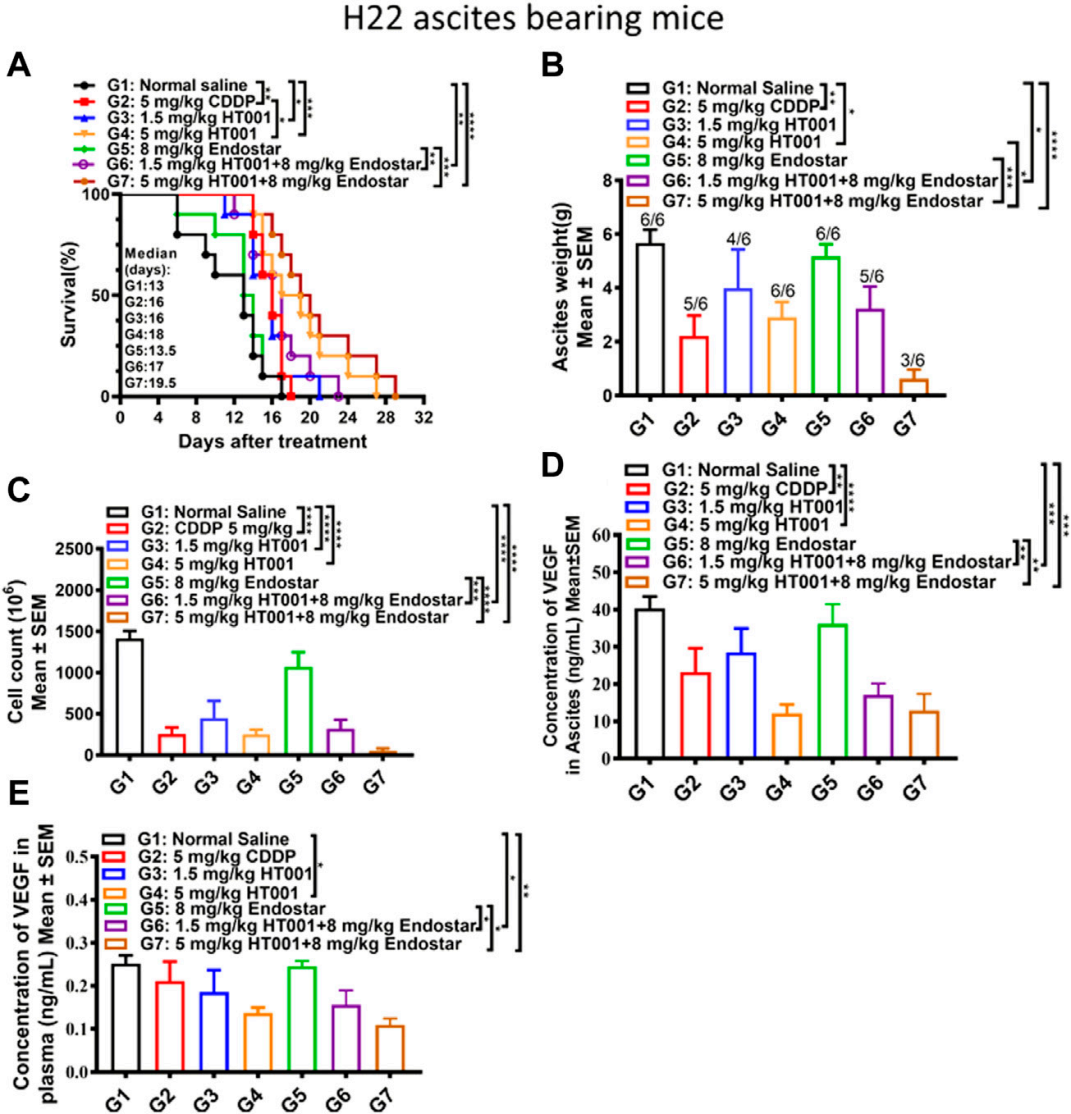
Effects of HT001 and Endostar combination on H22 ascites-bearing mice. The H22 bearing ascites mice were intraperitoneally administrated with normal saline, 5 mg/kg CDDP, 1.5 mg/kg HT001, 5 mg/kg HT001, 8 mg/kg Endostar, 1.5 mg/kg HT001+8 mg/kg Endostar, or 5 mg/kg HT001+8 mg/kg Endostar (*n* = 16/group). HT001 and CDDP were administrated once a week and Endostar was administrated daily for 3 weeks. After 8 days of administration, 6 mice from each group were sacrificed for analysis and the remaining mice were used for monitoring survival. **(A)** Kaplan-Meier survival analysis of H22 ascites-bearing mice. **(B)** Ascites weight of H22 ascites mice. The numbers on the column represent number of mice with ascites formed/all analyzed mice. **(C)** Cell counts in ascites of H22 ascites-bearing mice. **(D)** VEGF levels in ascites from H22 ascites-bearing mice. **(E)** VEGF levels in plasma from H22 bearing ascites mice. Error bars represent SEM. *, *p* < 0.05; **, *p* < 0.01; ***, *p* < 0.001; ****, *p* < 0.0001.

Due to potential linkages between tumor angiogenesis and ascites formation, the therapeutic effect of HT001 combined with Endostar was explored in the H22 ascites-bearing mice. As shown in Figure 6A, treatment with either CDDP or HT001 prolonged the survival of the H22 ascites-bearing mice similar to the effect seen in previous experiment. Combination of Endostar and HT001 further prolong the survival of mice. Accordingly, treatment of either CDDP or HT001 reduced ascites weight and cell counts while combination of Endostar and HT001 further inhibited ascites formation in mice (Figures 6B,C). Similar effects on VEGF reduction were also observed (Figures 6D,E). Thus, HT001 presented a synergistic anti-ascites efficacy in combination with Endostar in H22 ascites mouse model.

### *In vivo* pharmacokinetic profiles of HT001

**FIGURE 7:**
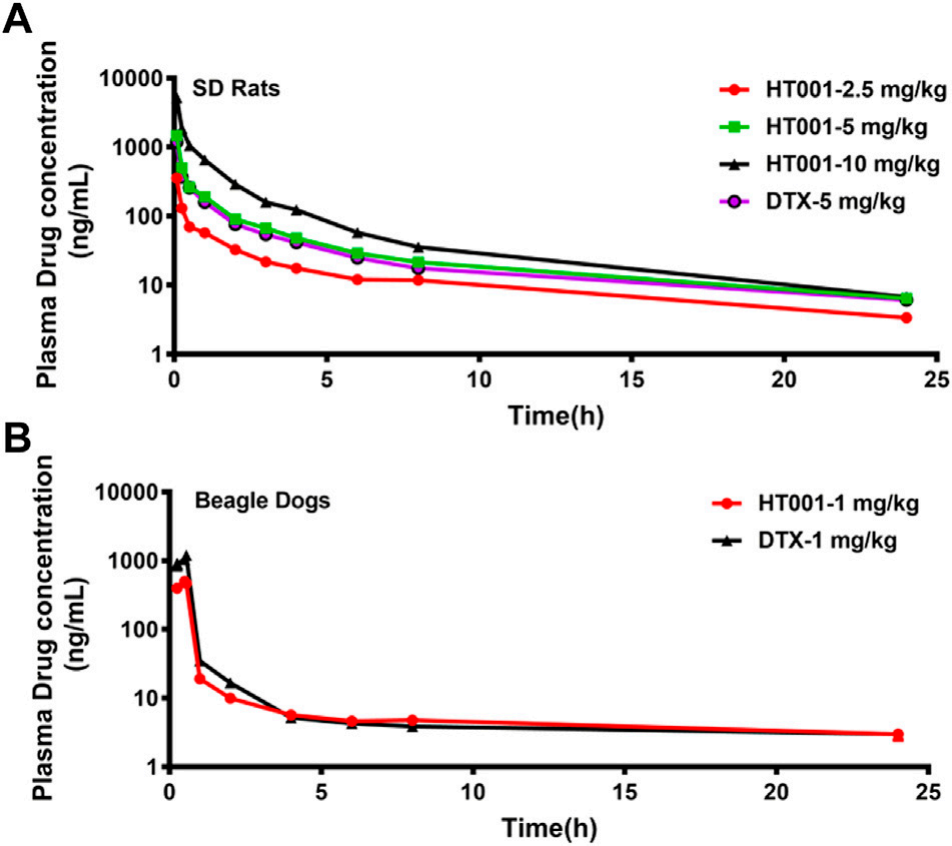
Plasma concentration-time curves of docetaxel in SD Rats and Beagle dogs. **(A)** SD rats were intravenously administered with a single dose of 2.5, 5, 10 mg/kg HT001 or 5 mg/kg DTX (six rats per group, male: female = 1:1) and plasma drug concentrations were analyzed by LC-MS/MS. **(B)** Beagle dogs were intravenously administrated with a single dose of 1 mg/kg of DTX or HT001 (six dogs per group, male: female = 1:1) and plasma drug concentrations were analyzed by LC-MS/MS.

The mean plasma concentration-time curves of docetaxel after administration with a single dose of HT001 or DTX via intravenous injection in SD rats are shown in Figure 7A. The PK parameters in SD rats are listed in Table 2. There was no significant difference in the main PK parameters between different genders in each group of rats (data not shown). In the dose range of 2.5–10 mg/kg of HT001, the values of C_max_ and AUC_last_ both increased with increasing dose, and the ratio of increased exposure was higher than that of increased dose. At a dose of 5 mg/kg, the AUC_last_ values of docetaxel in rats after DTX administration were significantly lower than that of HT001 treatment group.

**TABLE 2:** Pharmacokinetic parameters of HT001 and DTX in SD rats and Beagle dogs.

Animals	Group	t_1/2_ (h)	C_max_ (ng/mL)	AUC_last_ (h^∗^ng/mL)	AUC_inf_ (h^∗^ng/mL)	Vd (mL/kg)	Cl (mL/h/kg)	MRT (h)
SD Rats	HT001 (2.5 mg/kg)	10.15 ± 3.27	356.63 ± 73.66	378.34 ± 76.05	430.49 ± 72.53	88,065.11 ± 35,990.28	5940.07 ± 949.97	5.20 ± 0.66
	HT001 (5 mg/kg)	8.50 ± 1.35	1467.68 ± 336.31	1059.71 ± 148.37∗	1140.22 ± 170.57	54,080.35 ± 6302.53	4472.06 ± 703.77	3.80 ± 0.15
	HT001 (10 mg/kg)	6.10 ± 0.64	5235.81 ± 542.79	3049.33 ± 425.04	3108.82 ± 429.77	28,852.98 ± 5503.05	3270.65 ± 469.59	2.36 ± 0.06
	DTX (5 mg/kg)	9.28 ± 1.80	1191.14 ± 183.32	890.52 ± 98.35	972.95 ± 88.17	69,693.63 ± 16,608.23	5174.2 ± 467.60	3.93 ± 0.25
Beagle dogs	HT001 (1 mg/kg)	14.34 ± 8.04	500.81 ± 120.06∗∗∗∗	377.38 ± 88.33∗∗∗	449.49 ± 115.9∗∗∗	15,755.80 ± 9506.25∗	2358.70 ± 641.59∗∗	1.56 ± 1.02
	DTX (1 mg/kg)	19.45 ± 2.78	1256.36 ± 272.76	821.80 ± 179.51	913.21 ± 179.20	6709.52 ± 2370.26	1135.55 ± 251.82	1.12 ± 0.28

Abbreviations: t_1/2_, half-life; C_max_, maximum plasma drug concentration; AUC, area under curve; Vd, apparent volume of distribution; Cl, clearance; MRT, mean residence time.

Data were presented as Mean ± SD. *p*-value was calculated comparing HT001 and DTX groups at the same dose using the student’s *t* test; ∗, *p* < 0.05; ∗∗, *p* < 0.01; ∗∗∗, *p* < 0.001; ∗∗∗∗, *p* < 0.0001.

The mean plasma concentration-time curves of docetaxel after administration with a single dose of HT001 or DTX are shown in Figure 7B. The PK parameters in beagle dogs are listed in Table 2. There was no significant difference in the main PK parameters between different genders in each group of dogs (data not shown). At a dose of 1 mg/kg, the C_max_, AUC_last_, AUC_inf_, Vd, and Cl values of docetaxel after HT001 treatment were approximately half of that in the DTX treatment group. To be noted, allergic reactions, such as skin flushing, swelling and redness of the eyelids, and shortness of breath, were observed in each dog of DTX group during administration, although this phenomenon was not observed in HT001 group.

### Biodistribution and toxicity profiles of HT001

**FIGURE 8:**
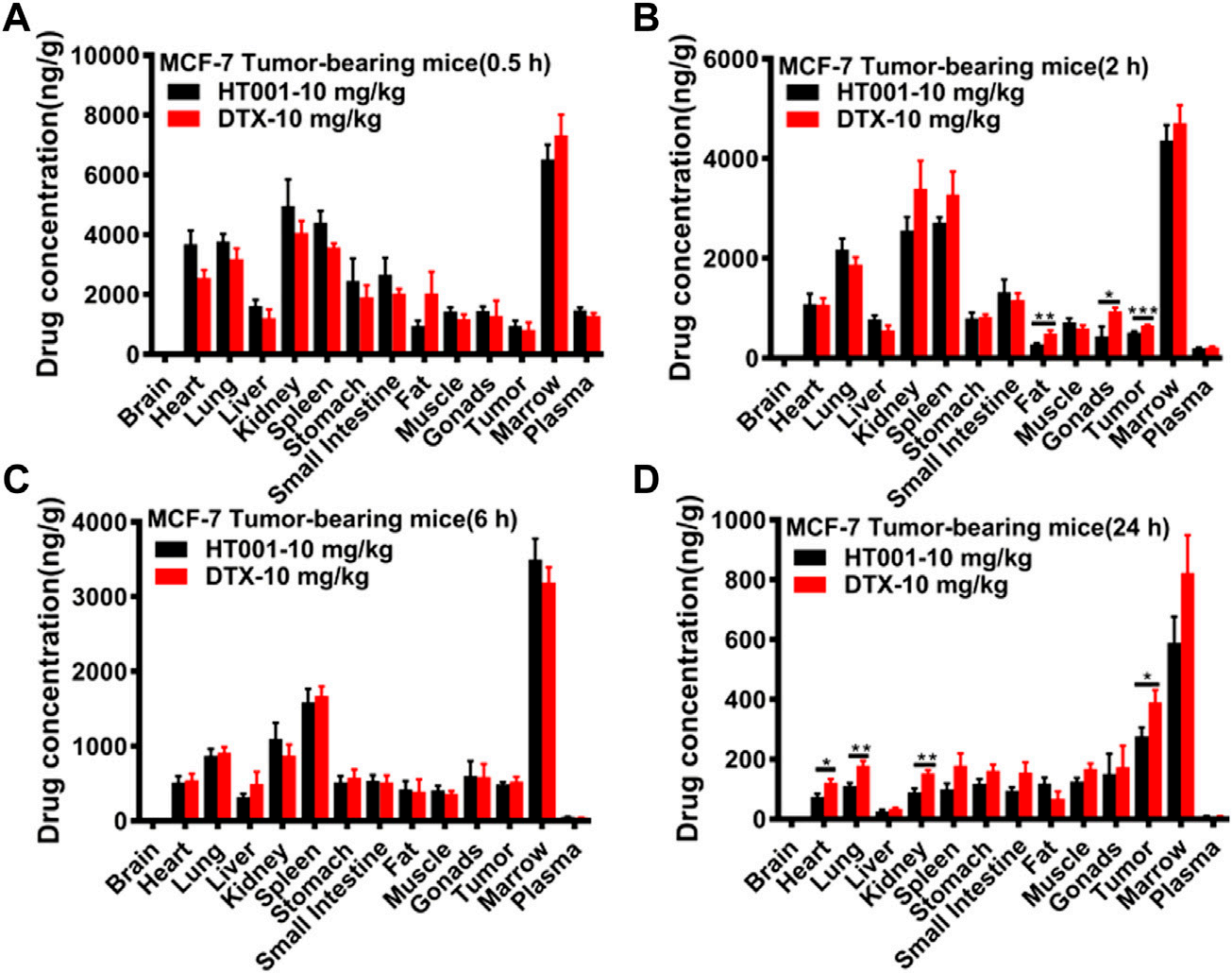
Tissue distributions of docetaxel in MCF-7 tumor-bearing mice. MCF-7 tumor-bearing Balb/c nude mice were intravenously administered with 10 mg/kg HT001 or DTX (*n* = 24, male: female = 1:1). **(A–D)** Drug concentration in plasma and different organs at each time point (0.5, 2, 6, and 24 h). Six mice from each group at each time point were sacrificed, and different organs were harvested to analyze the drug concentration by LC-MS/MS. *, *p* < 0.05; **, *p* < 0.01; ***, *p* < 0.001.

The *in vitro* drug release behavior of HT001 was described previously and HT001 had a significantly slower drug release than DTX when evaluated by a dialysis method (Zhang et al., 2022). To further investigate the drug release *in vivo*, biodistribution of docetaxel in major organs was assessed in MCF-7 tumor-bearing Balb/c nude mice at 0.5, 2, 6, and 24 h after intravenous injection of 10 mg/kg HT001 or DTX, and the results are illustrated in Figures 8A–D, respectively. At 0.5 and 6 h, the docetaxel biodistributions between the HT001 group and the DTX group were approximately the same. At 2 and 24 h, the docetaxel biodistributions were slightly lower in HT001 groups in some of the organs. Overall, similar trend and drug distributions in various tissues of tumor-bearing mice were observed between HT001 and DTX.

In tolerability studies in SD rats treated with multiple doses of HT001, all rats received low (5 mg/kg) and middle (10 mg/kg) doses tolerated well to the drug with no mortality. In high (16 mg/kg) dose group, the animals experienced 33% bodyweight loss and one rat died on the last day of the study. Pathological analysis revealed that in high dose group, an increased mitosis in liver hepatocytes and renal pelvis epithelium, a decreased white pulp lymphocytes population and an increased lymphocyte mitosis in the spleen were present, while these abnormal changes were rarely observed in middle dose-treated rats and not observed at all in low dose-treated animals (Supplementary Figures S2A–C). All rats received multiple doses of 5 mg/kg and 10 mg/kg tolerated well. The toxicological findings of 16 mg/kg dosing group are likely due to the very high dose level of the drug. This dosage (16 mg/kg) represents approximately 22-fold of the equivalent minimal efficacious dose, therefore, it will less likely be reached in clinical trials.

## Discussion

HT001 is a clinical stage and novel form of docetaxel polymeric micelles which is in Phase I clinical development in solid tumor patients. HT001 may have several lines of advantages in drug deliverability, pharmacology, and safety aspect. HT001’s excipient mPEG-PDLLA is amphiphilic biocompatible degradable material, with a critical micelle concentration (CMC) of 0.044 mg/ml and high solubility in water. The potential advantages of HT001 over its parental drug are as follows: 1) HT001 is formulated with safe excipient mPEG-PDLLA and has a significantly slower release (Kim et al., 2001; Deng et al., 2018; Yu et al., 2018; Zhang et al., 2022). mPEG-PDLLA is an auxiliary material with amphiphilic, biocompatible and degradable properties (Kim et al., 2001; Deng et al., 2018; Yu et al., 2018; Zhang et al., 2022). 2) Employment of new excipients avoids the clinical toxicity and side effects caused by Tween 80 and ethanol as co-solvents and significantly reduces allergic reactions. 3) Unlike DTX, HT001 can be administered in the clinic without pre-dosing of glucocorticoid, which simplifies clinical application and improves patient compliance. A recent preclinical study indicated that docetaxel polymeric micelles showed better efficacy with less systemic toxicity in ovarian cancer models (Zhang et al., 2022). The maximum tolerated dose (MTD) of HT001 and DTX are 100 mg/kg [internal data] and 30 mg/kg (Mishra et al., 2014) in mice, respectively, indicating HT001 may have a better safety profile *in vivo*. In this study, our toxicity data showed that in beagle dogs, allergic reactions were observed in DTX-treated group during administration, while this phenomenon was not observed in HT001 group. In addition, our pharmacology data reveal that, at least in some solid tumor xenograft mouse models, the anti-tumor effect of HT001 was superior to DTX and the bodyweight in HT001-treated mice was heavier than that of DTX group, when drugs were given at the same dose and frequency. These data, together with its acceptable preclinical PK and safety profiles, indicate that polymeric micelle formulated HT001 may have reduced toxic liability and potentially enhanced anti-tumor effects in comparison with DTX.

MA represents an unmet medical need lacking effective therapy in the market. MA originates from metastasis of malignant tumor cells to the peritoneum or peritoneal primary lesions and 50% of patients with advanced or recurrent malignant tumors develop MA in varying degrees (Sedláková et al., 1999). The survival of patients is usually around 5–7 months. A large volume of ascites is often an indicator of suboptimal cytoreduction, increased risk of disease recurrence, and associated with poorer progression-free and overall survival (Yoshizawa et al., 2000). Chemotherapy, in addition to systemic disease control, is a common first-line treatment for patients with ascites, while chemotherapy-induced drug toxicities place restrictions on its application. Although docetaxel is widely used in the treatment of solid tumors, it is not approved for malignant ascites. A case report showed that the liver tumor and ascites became undetectable when treated with 60 mg/m^2^ docetaxel in a primary duodenal cancer patient, which indicated the potential therapeutic effect of docetaxel in MA (Okada et al., 2013). However, docetaxel has poor aqueous solubility and systematic toxicity, bringing side effects that significantly affect patients’ quality of life. The combination of docetaxel with an integrative cancer care agent has been attempted in gastric cancer-derived MA and an encouraging response was achieved (Oh, 2020). In this study, we determined the effectiveness of a novel docetaxel micelle HT001 in 2 preclinical ascites formation mouse models as well as multiple solid tumor subcutaneous xenograft mouse models. Cisplatin was chosen as a reference compound as it is the most commonly used chemo drug for MA (Chu et al., 2017; Ishigami et al., 2018). In H22 and ES-2 ascites-bearing mice, HT001 suppressed the formation of ascites and prolonged the survival of ascites-bearing mice. Compared to Cisplatin group, HT001 showed a better efficacy at 5 mg/kg in both survival extension and bodyweight reduction (reflects reduced ascites) in these two ascites-bearing mouse models. Notably, the combination of HT001 with Endostar not only further reduced ascites production but also prolonged survival of H22 ascites-bearing mice. To our knowledge, this is the first report demonstrating anti-ascites formation activity and synergistic potency for a docetaxel polymeric micelle in combination with anti-angiogenic agents in MA treatment. These findings set scientific foundation and new direction for HT001 clinical development strategies to treat patients with MA.

Increased peritoneal microvascular permeability is the main factor contributing to the development of MA. VEGF is considered to be one of the critical factors that can stimulate vessel formation and promote vascular permeability, leading to formation of malignant metastases (Pereira et al., 2021). Given the fact that majority of tumors over express VEGF, anti-VEGF or anti-angiogenic modalities may have therapeutic potentials for the treatment of malignant ascites (Kobold et al., 2009). Previous investigations revealed encouraging responses of Bevacizumab when administered to the recurrent or chemotherapy-resistant ovarian cancer patients with symptomatic malignant ascites (Shimizu et al., 2019; Sjoquist et al., 2021). Endostar is an anti-angiogenic agent with multiple targets including the VEGF/VEGFR pathway and can enhance anti-tumor activities of some chemotherapy drugs and checkpoint inhibitors (Yao et al., 2017; Xue et al., 2022). A case report showed that when administered along with gemcitabine-cisplatin regimen, Endostar resulted in improvement of clinical symptoms and reduction of ascites in an advanced malignant mesothelioma patient (Chen et al., 2012), suggesting therapeutic potentials of Endostar to treat MA. Our data showed that HT001 not only suppressed ascites production but also prolonged overall survival of ascites-bearing animal models, with significantly reduced VEGF levels in both serum and ascites. Addition of Endostar into the regimen further enhanced therapeutic effect of HT001. Our study demonstrates the potential clinical utility of HT001 in combination with anti-angiogenic therapy in the treatment of malignant ascites. These findings provide scientific rationales to further explore the clinical applications and therapeutic values for both HT001 and Endostar to treat patients with MA.

The elevated levels of VEGF have been associated with several carcinomas including non-small cell lung cancer, breast cancer, gastric cancer, colorectal cancer and prostate cancer. High VEGF levels have been found to correlate with poor prognosis in gastric cancer- or ovarian cancer-derived MA (Fushida et al., 2013; Bhaskari and Krishnamoorthy, 2015). Our study showed that VEGF levels, in both serum and ascites, negatively correlated with anti-tumor efficacy and overall survival in multiple tumor models. These findings together with the published data suggest that serum and ascites levels of VEGF could serve as a predictive or prognostic biomarker in studies involving MA animal models or MA patients (Fushida et al., 2013; Bhaskari and Krishnamoorthy, 2015).

This study has limitations. Endostar is an anti-cancer agent targeting a broad spectrum of signaling pathways impacting biological events including angiogenesis, proliferation, mobility, and apoptosis of the cancer cells. Although the commercially available drug has been used in the clinic for decades, the MOA of endostatin is rather complex and is yet fully understood. Likewise, this study didn’t elucidate mechanistic interactions between endostatin and docetaxel polymeric micelles at molecular levels. More mechanistic studies dissecting not only the pharmacologic response, but also biologic effects of docetaxel micelles in combination with endostatin or with other anti-angiogenic inhibitors (Xue et al., 2021; Zhou et al., 2021), are required.

## Conclusion

This study demonstrates that HT001 is an effective anti-tumor agent in several solid tumor and malignant ascites animal models with a good safety profile. Notably, the combination of HT001 with Endostar, a recombinant human Endostatin, reveals significantly synergistic anti-ascites effects in a liver cancer ascites mouse model. These data suggest that HT001 might be a promising chemotherapy agent with a clinical development value as monotherapy and in combination with anti-angiogenic therapy. HT001 will be investigated in clinical studies involving cancer patients with MA.

## Data Availability

The original contributions presented in the study are included in the article/Supplementary Material, further inquiries can be directed to the corresponding authors.
