# Supplementary material for: A novel form of docetaxel polymeric micelles demonstrates anti-tumor and ascites-inhibitory activities in animal models as monotherapy or in combination with anti-angiogenic agents
Source: Front Pharmacol. 2022 Aug 24;13:964076. doi: 10.3389/fphar.2022.964076 (PMC9449419; doi:10.3389/fphar.2022.964076)
Supplement: Supplementary file 1 [file DataSheet1.docx]

Supplementary Material

# Supplementary Figures


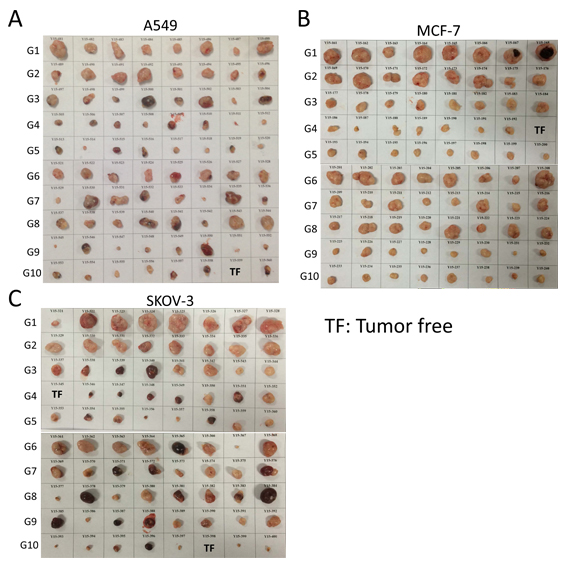


**Supplementary Figure 1.** Anti-tumor effects of HT001 and DTX in A549, MCF-7, and SKOV-3 xenograft mouse models. A549, MCF-7, and SKOV-3 tumor-bearing mice were intravenously administrated with normal saline, DTX, or HT001 once a week or once every 2 weeks (n=8/group). **(A-C).** Tumor photographs of each group on experimental end point in A549, MCF-7, and SKOV-3 tumor-bearing mice. TF: Tumor free.


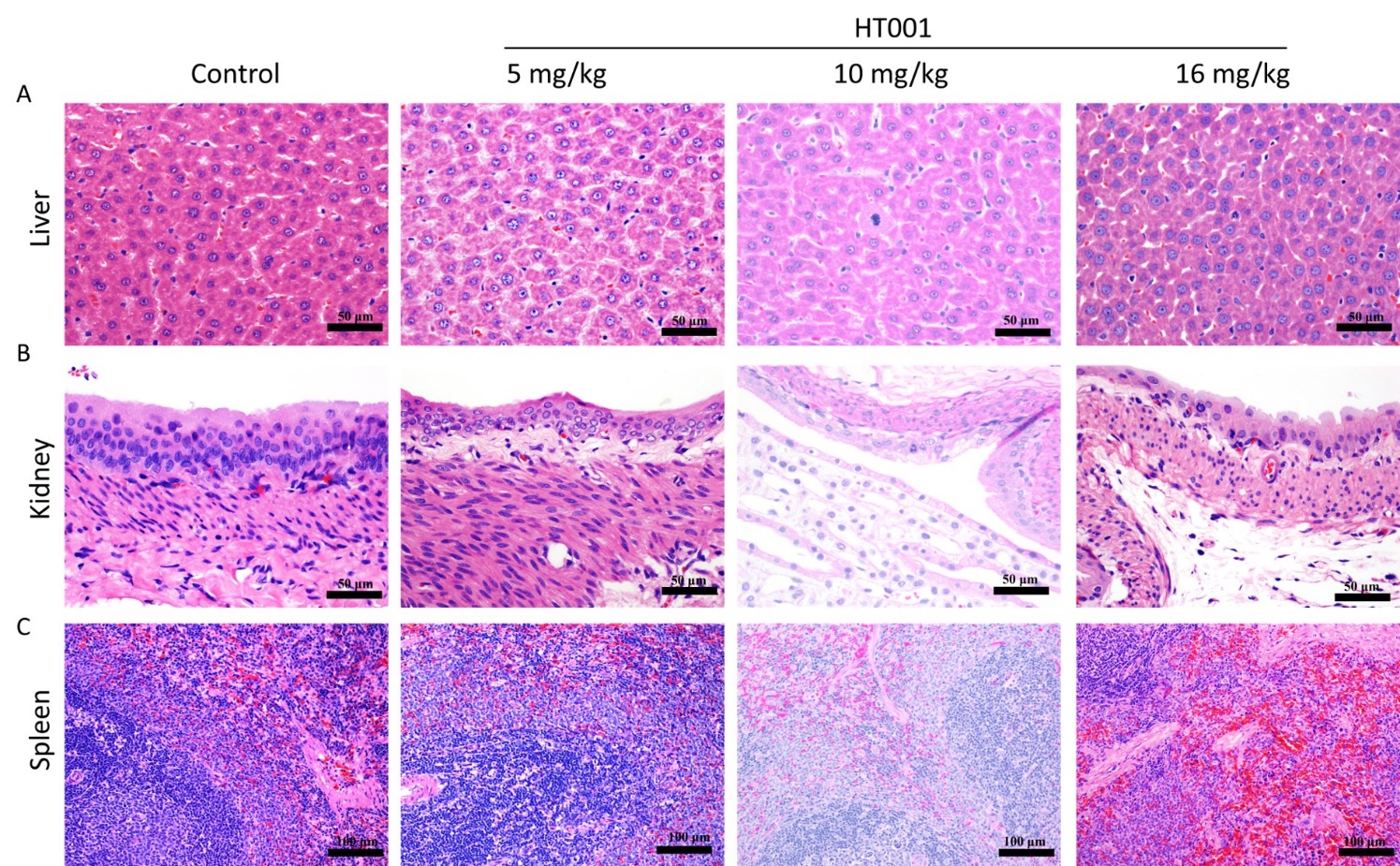


**Supplementary Figure 2.** Hematoxylin and Eosin-stained sections of the liver, kidney, and spleen tissue of rats treated with HT001. SD rats were intraperitoneally administered with normal saline, 5, 10, or 16 mg/kg HT001 once a week (n=10/group). On the day after the fifth dose, rats were anesthetized and sacrificed. The liver, kidney, and spleen samples were harvested and stained using the Hematoxylin and Eosin method. **(A-C)**. The representative images of liver, kidney, and spleen are shown. Scale bars represent 50 μm in liver and kidney, 100 μm in spleen.
